# Report of the Cases Treated in the Male Medical Ward of the Buffalo Hospital of the Sisters of Charity, Austin Flint, M. D., Attending Physician, during the College Session of 1856—7, Viz. from Oct. 1, 1856, to Feb. 18, 1857

**Published:** 1857-04

**Authors:** A. W. Nichols

**Affiliations:** Assistant to Chair of Clinical Medicine, University of Buffalo


ART. IV.—*Report of the Cases treated in the male medical ward of the Buffalo Hospital of the Sisters of Charity, Austin Flint, M. D., Attending Physician, during the College Session of* 1856—7, *viz. from Oct.* 1, 1856, *to Feb.* 18, 1857. Reported by A. W. NICHOLS, M. D., Assistant to Chair of Clinical Medicine, University of Buffalo.

Thirty-seven different diseases have been treated, and one hundred and twenty-eight cases of disease, (see tabular statement.) The number of patients treated is one hundred and seventeen; of this number, sixty have either been discharged as cured or remain in hospital free from disease; forty report very much improved; ten have died,—and the remainder are either not improved or the disease is still progressive. (See table.)

Of the fatal cases, two were from typhoid fever; one from cirrhosis of the liver; one from Bright's disease, and six from pulmonary tuberculosis. The patient with cirrhosis was affected with ascites, and was tapped repeatedly. The patient who died with Bright's disease, was attacked in the hospital with pericarditis. One of the patients with tuberculosis, had chronic pleurisy; another had sub-acute rheumatism; and two others were in the advanced stages of tuberculosis when admitted.

Many interesting points were presented pertaining to the various forms of disease which occurred in the ward; a few of them are noticed below, in connection with the diseases in which they were observed.*

Thus, of the twenty-four cases of *intermittent fever*, three were of the double quotidian type; one case of intermittent fever, was preceded and followed by intercostal neuralgia; one case followed typhoid fever; another case preceded and followed typhoid fever.

Of the seven cases of *typhoid fever*, one was preceded by epilepsy and followed by an epileptic paroxysm and insanity; two cases presented parodi-tis; one of these patients died and one recovered.

---

* We are happy to inform our readers that detailed reports of some of the cases under observation at the hospital during the late session, from the pen of Dr. Nichols, will appear in subsequent numbers of this Journal.—ED. BUFFALO MED. JOUR.

Of the nine cases of *rheumatism and gout*, one was acute articular, complicated with a mitral bellows-murmur; five cases were sub-acute; and two chronic.

Of the seven cases of *pneumonitis*, two were latent; one was complicated with pulmonary tuberculosis; and three proceeded to a favorable termination without any medication.

Of the fifteen cases of *pulmonary tuberculosis*, one was affected with pneumonitis; one had empyema; one was laboring under chronic pleurisy; one presented caries of the spine and angular curvature; and two had attacks of sub-acute rheumatism.

Of the three cases of *emphysema*, one was uncomplicated with bronchitis; one was accompanied by asthmatic paroxysms, which were promptly relieved by the inhalation of chloroform.

Of the four cases of *dysentery*, all were treated with saline cathartics, and afterward with opiates, and all recovered speedily.

Of the four cases of *albuminuria*, one had amaurosis, and pericarditis supervened; effusion occurred in both pleural sacs, and death took place by sudden coma.

Of the four cases of *intercostal neuralgia*, three cases presented the three points of tenderness, well marked; and in one case, the middle point of tenderness alone was present.

The five cases of *favus* presented the characteristic cryptogamous plant, under the microscope; four were treated with cod liver oil, externally.

The mortality was one in twelve, nearly; average of cured, about one-half; average improved, about one-third.

Clinical lectures have been given upon twenty-eight different diseases, including their symptomatology, diagnosis, pathology, and treatment.

These lectures have included the following subjects, viz: Dysentery, cirrhosis, ascites and anasarca, icterus, colica saturnina, intermittent and typhoid fevers, rheumatism and rheumatic gout, favus, scabies, intercostal neuralgia, hemiplegia, arcus senilis and apoplexy, pericarditis, valvular lesions of heart, asthma, emphysema, pneumonitis, pulmo-tuberculosis, pleurisy-chronic, empyema, *pneumo-thorax*, together with a few other diseases.

*Tabular Statement.*

| NAME OF DISEASE. | Total Number. | Discharged cured. | Discharged improved. | Discharged not improved. | Cured and remaining in hospital. | Improved and remaining in hospital. | In hospital and not improved. | In hospital and disease progressing. | Died. |
|---|---|---|---|---|---|---|---|---|---|
| Fever Intermittent, - - - | 24 | 19 | 3 | | | 2 | | | |
| " Typhoid, - - - - | 7 | 4 | | | 1 | | | | 2 |
| Gout and Rheumatism, - - - | 9 | | 3 | 1 | | 4 | | | 1* |
| Bronchitis, - - - - - - | 4 | 2 | | | | 2 | | | |
| Pneumonitis, - - - - - - | 7 | 5 | 1 | | 1 | | | | |
| Pulmo-Tuberculosis, - - - | 15 | | 5 | | | 1 | | 3 | 6 |
| Emphysema, - - - - - - | 3 | | | | | 1 | 2 | | |
| Pleurisy, chronic, - - - | 2 | 1 | | | | | | | 1† |
| Empyema, - - - - - - | 1 | | | | 1 | | | | |
| Pneumo-Thorax, - - - - | 1 | | | | | 1 | | | |
| Pericarditis, - - - - - | 1 | | | | | | | | 1‡ |
| Valvular Disease, Heart, - - | 2 | | | | | 1 | 1 | | |
| Dilatation of Stomach, - - - | 1 | | 1 | | | | | | |
| Diarrhœa, - - - - - - | 9 | 9 | | | | | | | |
| Dysentery, - - - - - - | 4 | 4 | | | | | | | |
| Icterus, - - - - - - | 1 | 1 | | | | | | | |
| Cirrhosis and Ascites, - - - | 1 | | | | | | | | 1 |
| Anasarca, - - - - - - | 1 | | | | | 1 | | | |
| Albuminuria, - - - - - | 4 | | 1 | 1 | | 1 | | | 1§ |
| Incontinence of Urine, - - - | 1 | | | | | 1 | | | |
| Epistaxis, - - - - - - | 1 | 1 | | | | | | | |
| Colica Saturnina, - - - | 2 | 2 | | | | | | | |
| Neuralgia, - - - - - - | 2 | | 1 | | | 1 | | | |
| Intercostal Neuralgia, - - | 4 | 2 | | | | 1 | 1 | | |
| Delirium Tremens, - - - | 1 | 1 | | | | | | | |
| Delirium Ebriosum, - - - | 1 | 1 | | | | | | | |
| Insanity, - - - - - - | 1 | | | | | | 1 | | |
| Hemiplegia, - - - - - | 2 | | | | 2 | | | | |
| Epilepsy, - - - - - - | 2 | | | 1 | | | 1 | | |
| Pseudo-apoplexy with arcus senilis, | 1 | | 1 | | | | | | |
| Tuberculous Disease of Spine, - | 1 | | | | | 1 | | | |
| Syphilitic Disease of do. - | 1 | | | | | 1 | | | |
| Favus, - - - - - - - | 5 | 1 | | | 1 | 3 | | | |
| Eceyema, - - - - - - | 1 | | | | | 1 | | | |
| Scabies, - - - - - - | 3 | 1 | | | | 2 | | | |
| Icthyosis, - - - - - - | 1 | | | 1 | | | | | |
| Convalescence, typhoid fever,- - | 1 | | | | | 1 | | | |
| Totals, - - - - - | 128 | 54 | 16 | 4 | 6 | 26 | 6 | 3 | 10 |

* Died of tuberculosis and included in the six deaths from that disease.

† The case of chronic pleurisy, also, included among six deaths.

‡ Pericarditis occurring in course of albuminuria or Bright's disease.

§ This case was complicated with pericarditis.

